# Supplementary material for: Astrocytes-Derived Small Extracellular Vesicles Hinder Glioma Growth
Source: Biomedicines. 2022 Nov 17;10(11):2952. doi: 10.3390/biomedicines10112952 (PMC9688032; doi:10.3390/biomedicines10112952)
Supplement: Supplementary file 1 [file biomedicines-10-02952-s001.zip › biomedicines-1776536-supplementary.pdf]

Supplementary Figure S1

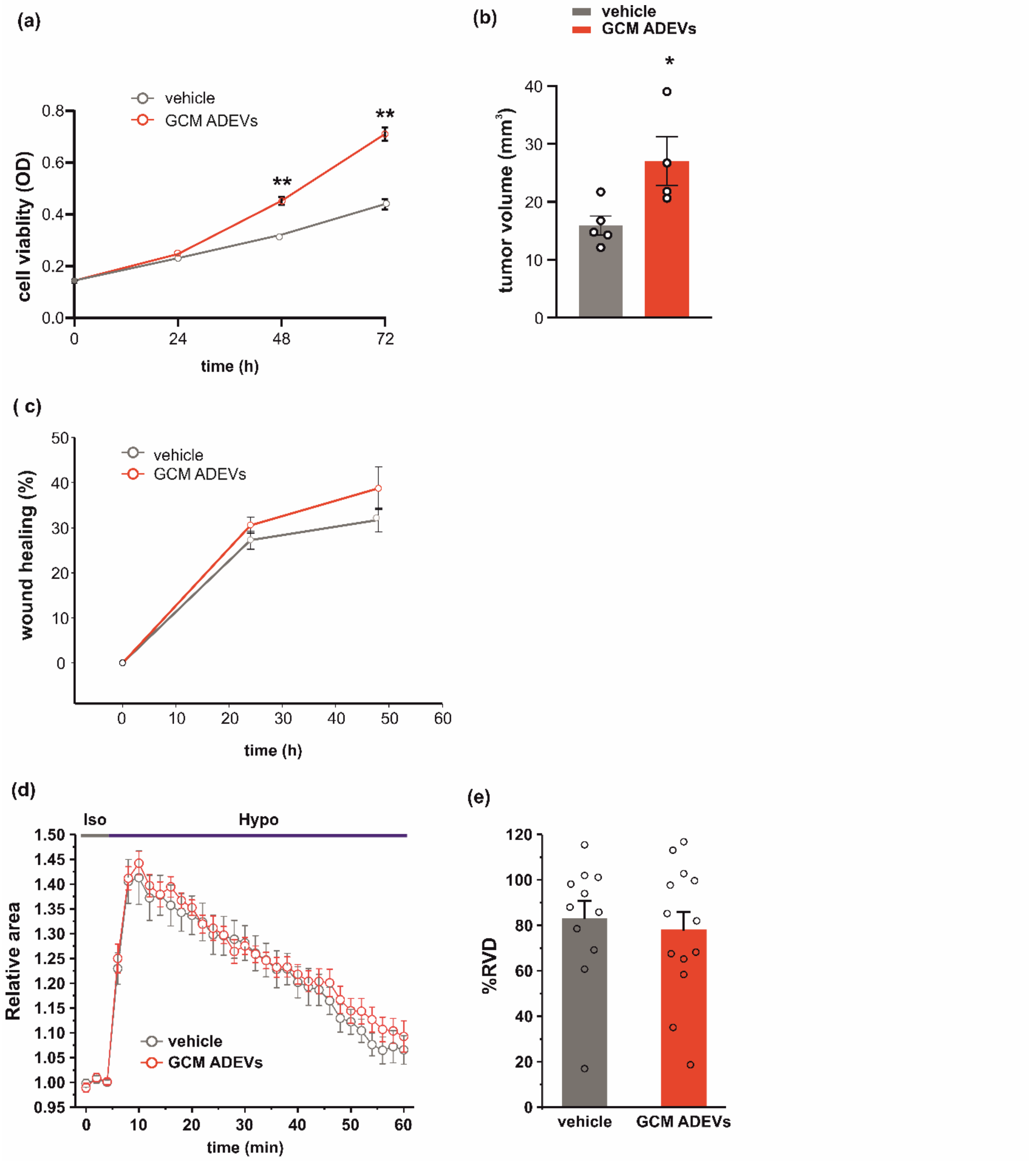

Supplementary Figure S1. GCM ADEVs modulate glioma cells. (a) GL261 cells were assayed for cell viability with sEV-free fraction medium (vehicle) or in presence of ADEVs derived from astrocytes treated with glioma-conditioned medium, GCM (GCM ADEVs) by MTT analysis. Cells were analyzed 0, 24, 48 and 72 h after plating. Viability was reported in mean optical density (OD) ± SE, N = 4; Student's t-test, $^{**}p \leq 0.001$. (b) Tumor size in the brain of GL261-bearing mice treated with sEV-free fraction medium (vehicle) or GCM ADEVs. Tumor size (in mm^3) is reported as mean ± SE, N = 4-5/experimental group, Student's t-test, $*p < 0.05$. (c) GL261 glioma cells were treated with vehicle or GCM ADEVs and a wound healing (migration) assay was performed. GL261 migration was measured 24 and 48 h after treatment, data are expressed as mean percentage of wound healing area ± SE, N = 3, Student's t-test, ns. (d) Time course of RVD as evaluated from the changes of the relative cell area (Arel) in vehicle-treated cells (Iso, grey bar) and during application of 30% hypotonic solution (Hypo, blue bar) to GL261 vehicle-treated cells (vehicle, grey circles) and to GCM ADEVs-treated GL261 cells (GCM ADEVs, red

circles). (e) Bar plot showing the average percentage of RVD in vehicle- and GCM ADEVs-treated GL261 cells, calculated as follows: $\%RVD = (A_{rel,peak} - A_{rel,56\ min})/(A_{rel,peak}))*100$. Data are shown as mean ± SEM. N = 3, Student’s t-test, ns.
